# Chitosan-Based Biomaterial in Wound Healing: A Review

**DOI:** 10.7759/cureus.55193

**Published:** 2024-02-28

**Authors:** Suba Rajinikanth B, Densingh Samuel Raj Rajkumar, Keerthika K, Vinothini Vijayaragavan

**Affiliations:** 1 Pediatrics, Faculty of Medicine, Sri Lalithambigai Medical College and Hospital, Chennai, IND; 2 Traumatology and Orthopedics, Kursk State Medical University, Kursk, RUS; 3 Biotechnology, ACS Advanced Medical Research Institute, Dr MGR Educational and Research Institute, Chennai, IND

**Keywords:** chitin, biopolymer, scaffolds, hydrogels, nanoparticles, wound healing, biomaterials, chitosan

## Abstract

Wound healing is an evolving and intricate technique that is vital to the restoration of tissue integrity and function. Over the past few decades, chitosan a biopolymer derived from chitin, became known as an emerging biomaterial in the field of healing wounds due to its distinctive characteristics including biocompatibility, biodegradability, affinity to biomolecules, and wound-healing activity. This natural polymer exhibits excellent healing capabilities by accelerating the development of new skin cells, reducing inflammation, and preventing infections. Due to its distinct biochemical characteristics and innate antibacterial activity, chitosan has been extensively researched as an antibacterial wound dressing. Chronic wounds, such as diabetic ulcers and liver disease, are a growing medical problem. Chitosan-based biomaterials are a promising solution in the domain of wound care. The article analyzes the depth of chitosan-based biomaterials and their impact on wound healing and also the methods to enhance the advantages of chitosan by incorporating bioactive compounds. This literature review is aimed to improve the understanding and knowledge about biomaterials and their use in wound healing.

## Introduction and background

Biomaterials are a class of materials that deal with biological systems, including active cells, tissues, and bodily systems. They play an important contribution in many medical, pharmaceutical, and biotechnological applications. The primary purpose of biomaterials is to either replace or augment biological functions, often within the human body. The recent therapeutic usage of biomaterials in wound healing has gained momentum and products are being under trial. The fundamental character of biomaterials in the engineering of tissue is to offer structural assistance and weight transfer in order to stimulate cell attachment, expansion, and separation, as well as to regulate the form and size of the restored tissue.

In addition, biomaterials, often called scaffolds, can emit signals with spatial and temporal precision; this is critical for modifying cell performance and productivity and driving optimal regeneration of tissue. At present, there are numerous options for constructing a particular biomaterial that will eventually get utilized as a scaffold, involving organic biomaterials, synthetic biomaterials, and composites made up of a variety of materials [1].

The most commonly used to make wound dressings are biopolymers and synthetic polymers. Chitosan is a natural biopolymer obtained from chitin that can be observed within the shells of crustaceans like shrimps and crabs, as well as in the cell walls of some fungi. It is formed by deacetylating chitin, a process that involves removing the acetyl groups from chitin molecules. Chitosan’s versatility as well as distinctive features resulted in its widespread adoption within various sectors, making it valuable and sustainable with extensive varieties of possibilities.

A wound can be described as any sort of harm to living skin or tissue. Wounds are sometimes referred to as injuries, scars, cuts, and bruises depending on the injury components. Acute and chronic wounds fall into two categories. The type, size, and depth of an injury influence how quickly it heals. Acute wounds can recover between 60 to 90 days. Chronic wounds heal considerably more slowly, won't go across every phase of the typical wound healing process, and typically stop at the inflammatory stage, due to existing co-morbid conditions [2]. Chitosan-based wound dressings establish an ideal atmosphere for wound healing, by maintaining moisture, promoting cell migration, and reducing inflammation. As a result, chitosan is being explored for a variety of medical uses including implant coatings, tissue engineering, and for the administration of drugs and genes.

The main drawback of the currently available synthetic dressings is that they get adherent to lesions, which necessitates mechanical debridement and is painful for the patient, and also harmful to the freshly formed granulation tissue. Whereas, the biodegradable chitosan-based wound dressings are easily absorbed through the epidermis through the re-epithelization process, which avoids any need for mechanical debridement and injury to healthy granulation tissue [3]. This literature review is aimed to improve the understanding and knowledge about biomaterials and their use in wound healing.

Chitosan is a non-toxic, biodegradable polymer with excellent biocompatibility, minimal immunological reactions, mucoadhesive qualities, and features that promote absorption. Due to chitosan’s outstanding biological properties, which include stimulating blood coagulation, fibroblast proliferation, and collagen deposition, wound healing can be enhanced. Chitosan-based biomaterials continue to be an area of active research and development with the potential to address various medical and healthcare challenges. Their biocompatibility, biodegradability, and antimicrobial properties make them valuable components in the field of biomaterials. Chitosan is an effective choice for a biomaterial since it is versatile and can easily be adjusted to change its structure or properties [4].

## Review

Biomaterials

Definition

Biomaterials are substances that can or have been created that assume a form that, either by themselves or in conjunction with other intricate structures, can be utilized to direct the progress of any medical or clinical treatment in human or animal health care by regulating the interaction with the biological system's constituent parts [4]. It may be a substance or material that is used in the development of a medical instrument to interrelate with human tissues in order to monitor the functions of the body or to treat pathological disorders in the body [5]. Biomaterials may be natural or synthetic and are employed in medical applications to assist, augment, or restore damaged tissue or a biological function [6].

Types

Based on various properties, biomaterials can be distinguished into three basic groups: metal biomaterials, ceramic biomaterials, and polymer biomaterials.

**Figure 1:**
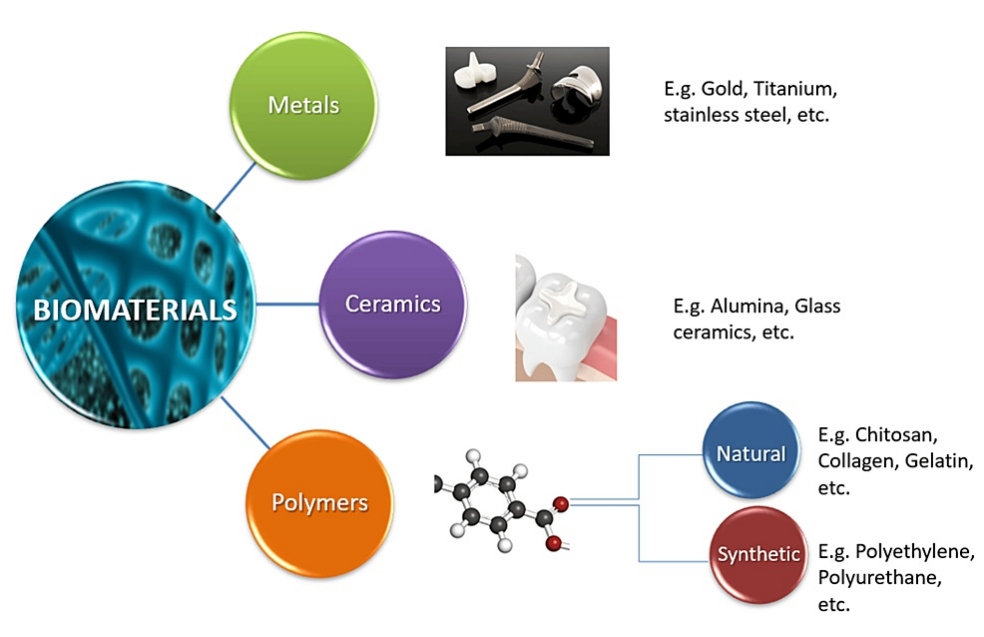
A schematic representation of the classification of biomaterials

The primary categories of sustainable biomaterials are natural polysaccharides, such as chitosan and pectin, and proteins like collagen and fibrin [7]. A schematic representation of the classification of biomaterials is shown in Figure 1.

In biomedical equipment, metallic biomaterials are frequently used because of their mechanical and biological properties. They find application in the field of orthopedics because of their resilience to fractures and resistance to tensile and shear loads. Their chemical bio-tolerant property allows biomedical alloys to communicate with their surrounding structures and release charged particles at harmless levels. Additionally, they are bio-inert, which limits the chemical interactions they have with surrounding cells, though a capsule of fibrous material could develop surrounding it. Historically, such compounds have been utilized in dental implants, orthodontic wires, and knee and hip prostheses [8].

Ceramic biomaterials outperform metal-based biomaterials in mechanical properties, plasticity, biocompatibility, melting temperature, and non-corrosivity. Ceramic biomaterials lack bone's high fracture toughness and elastic modulus are brittle and hard in comparison to bone [9].

Polymeric biomaterials are biocompatible, flexible, and can be easily fabricated. It can be classified into synthetic (e.g., poly lactic-co-glycolic acid {PLGA}, polyanhydrates), natural (e.g., hyaluronan, chitosan), and inorganic polymers (hydroxyapatite). Polymeric biomaterials can be coupled with other materials as composites for their mechanical, electrical, chemical, and thermal characteristics [10,11]. Numerous synthetic polymers, including poly-L-lysine, poly lactic-co-glycolic acid, polycaprolactone, and others are used as drug delivery vehicles [12].

Natural polymers possess biocompatibility and minimal immunogenicity and have long been employed in the fabrication of biomaterials. Synthetic polymers are ideal solutions for patients who are allergic to organic polymer compounds since they are less susceptible to antibodies and do not trigger persistent immunological inflammatory conditions. Synthetic polymers also provide more durability than natural polymers [13].

Chitin and chitosan

Composition

The semi-crystalline polysaccharide chitin, which is one of the most prevalent natural substances, is made up of monomeric units of - (1→4)-2-amino-2-deoxy-D-glucose and - (1→4)-2-acetamide-2-deoxy-D-glucose. They are an organic polymer that comprises monomers of glucosamine and N-acetylglucosamine joined together by -(­1­­­­­→4)-glycosidic linkages [14].

N-acetyl-D-glucose amine and D-glucose amine are copolymers to form chitosan. Because chitosan molecules contain both amino and hydroxyl groups, they can etherify, esterify, and reductively amate to generate stable covalent bonds. Chitosan is a deacetylated form of chitin that contains at least 50% free amine. It is frequently made from the main component of crustacean exoskeletons, such as shrimps, and has a structure of 2-acetamido-2-deoxy-D-glucopyranose and 2-amino-2-deoxy-D-glucopyranose units [15].

Properties

Chitin and chitosan are both widely employed in biomaterials studies due to their different functional properties. Chitin and chitosan are currently receiving a great deal of interest for usage in medical and pharmaceutical fields because of their distinct chemical and biological properties.

Native chitin possesses intriguing characteristics like gel-forming abilities, biodegradability, non-toxicity, and antimicrobial properties. Its average molar weight is often larger than 106 Daltons. Chitin has properties that are helpful for promoting quick skin regeneration and quick wound healing [14].

Chitosan is an alkaline polysaccharide that occurs naturally and is sustainable. It has excellent moisturizing as well as adsorption qualities without being poisonous or having any negative side effects [16]. Chitosan has a cationic nature because the amino group (-NH2) is present in its molecular makeup. The positive charge helps to generate an extracellular matrix by attracting negatively charged molecules to it. A hydroxyl group (-OH) is also included in the structure, which draws positively charged molecules to the structure to reinforce the interaction. In addition to electrostatic attraction, these functional groups help change chitosan, boosting its physical and biochemical properties to provide special characteristics and potential uses.

Chitosan dissolves in acid solutions, allowing it to react with other useful materials to form composites [17] and could be used to easily create scaffolds, membranes, gels, nanofibers, microparticles, and sponges [18].

Chitosan contains a wide range of biological actions and health advantages, including the ability to reduce the occurrence of gastric ulcers which is anti-inflammatory, protects against genotoxic effects, fights cancer, among others [19], and also positively impacts quicker recovery in various stages of wound healing including fibroplasia and collagen synthesis [20].

The capacity of chitooligosaccharides (CHOSs) for cell migration enhances the tissue remodeling stage of the healing process for wounds [21]. Chitosan hydrogels aid in fibroblast cell growth to expedite wound healing [22].

Antimicrobial activity

Due to its innate antibacterial action, chitosan among the many alternatives exhibits tremendous potential in antimicrobial application.

Antibacterial Activity

Gram-negative bacterial cell walls are characterized by hydrophilicity and negative charge than gram-positive bacterial ones. As a result, chitosan demonstrated a higher contact with gram-negative bacteria, which increased its antibacterial efficacy against them. Chitosan's antibacterial properties help to reduce the possibility of spreading disease and the occurrence of problems that slow down wound healing.

The chitosan dressing improved the beneficial bacteria *Prevotella*, *Lactobacillus*, and *Oscillibacter*, which support skin defense and recovery from injuries. It also shielded the wound from possible infection by bacteria like staphylococci, Enterococcus spp., Enterobacter spp., and Parabacteroides. These findings suggest that chitosan coating promotes coagulation and acts as a powerful antimicrobial, but it also promotes the healing of injuries by feeding beneficial microorganisms. As a result, chitosan covering may be appropriate as a primary technique for treating infections [23].

Gram-negative bacterial cell walls have higher levels of hydrophilicity and negative charge than gram-positive bacterial ones. As a result, chitosan demonstrated a higher contact with gram-negative bacteria, which increased its antibacterial efficacy against them [24].

Anti-inflammatory Activity

The structural components of chitosan's molecule, which are independent of MV (viscosity-average molecular weight), are what contribute most to the anti-inflammatory effect of the substance [25]. Chitosan supports the treatment of acid indigestion and peptic ulcers because it is alkaline and has amino acid groups that are free which can eliminate digestive acids and produce a barrier of protection in the intestines. The glucosamine hydrochloride or its phosphate, sulfate, and other salt production through salt transformation, as well as the acid hydrolysis of chitosan, are accountable to the anti-inflammatory mechanism of chitosan [26].

Anti-oxidant Activity

Chitosan and its derivatives function as antioxidants by killing extremely persistent DPPH radicals used in experimental studies as well as oxygen radicals including, superoxide, hydroxyl, and alkyl. Low molecular weight chitosan samples showed a greater capacity to scavenge various radicals. Bioadhesive and bacteriostatic chitosan also functions as an antioxidant, chelating agent, and hemostatic agent [27].

Anti-tumor Activity

Through both in vitro and in vivo testing, the intrinsic anticancer activity of chitosan and its low molecular weight variants was confirmed [28]. Chitosan has antitumor effects against a number of tumor cell strains under laboratory conditions and can be used in tumor-fighting due to the high molecular weight of chitosan's biodegradability and biocompatibility. Different molecular weights of chitosan and degree of deacetylation (DDA) have been linked to growth-inhibitory actions toward tumors in clinical mice, while their anticancer action seems to rely upon their chemical structure and molecular size [29].

Phases of wound healing

The several stages that often constitute the wound healing process are hemostasis, inflammation, proliferation, and remodeling. The inflammatory phase is characterized by hemostasis and inflammation, which occurs when collagen that has been exposed as a result of wound development and blood clot components release cytokines and growth factors to initiate the inflammatory phase [30].

**Figure 2:**
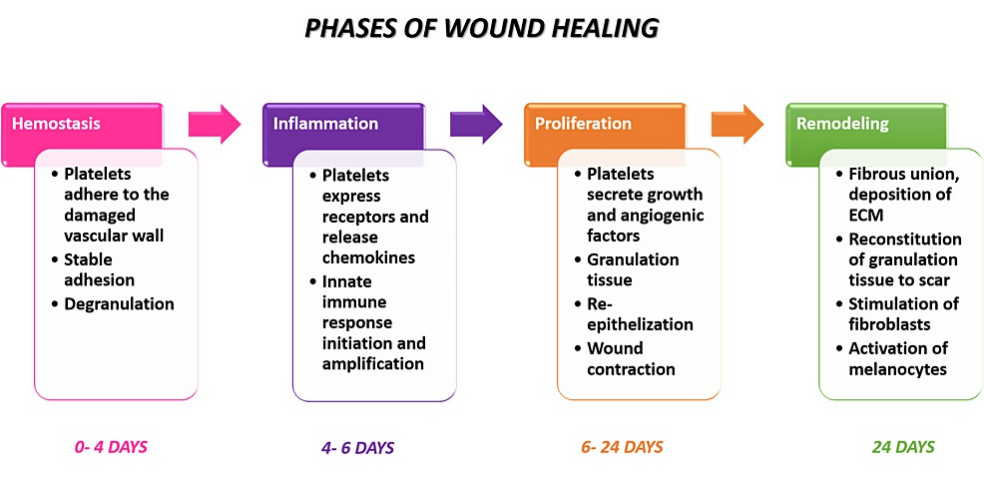
A schematic representation of the phases of wound healing ECM: Extracellular matrix

The key process in the healing of wounds is the dynamic shift from the inflammatory phase to the proliferative phase. The (a) fibroplasia, (b) re-epithelialization, (c) epithelial-mesenchymal interaction, (d) angiogenesis, (e) peripheral nerve repair are all signs of the proliferative phase and it is orchestrated by macrophages, predominant in inflammatory phase [31]. The schematic representation of different phases of wound healing is illustrated in Figure 2.

At the completion of the granulation tissue formation, the remodeling phase begins [32]. The immature scar might proceed to the ultimate remodeling stage once the wound has healed. Depending upon the wound severity, the remodeling phase could last up to a year before the wound starts to heal properly [33].

Role of chitosan in wound healing

The complex and highly coordinated wound healing process takes place in the four stages of hemostasis, inflammation, proliferation, and skin remodeling.

Hemostasis

Hemostasis is the initial phase of wound healing that begins as soon as an injury occurs. Its primary goal is to control bleeding and prevent excessive blood loss. Blood vessels tighten (vasoconstriction) to limit blood flow and prevent excessive blood loss.

Erythrocyte accumulation and fibrinolysis are both prevented by chitosan, which also encourages platelet adhesion and aggregation [34]. The reactions among positively charged ions in chitosan and negatively charged ions in red blood cell membranes may alter biological responses to proteins. These interactions could improve hemagglutination and promote the adherence of platelets and stimulation [35].

Chitosan possesses a strong positive charge due to its amino group (-NH2), which helps it to attract and bond with negatively charged constituents such as RBCs at the wound site, and when combined with a procoagulant, it forms a blood clot to stop bleeding quickly.

Inflammatory Phase

Inflammation, which appears as redness, heat, swelling, and pain at the injury site, is an important stage in the course of wound healing. It includes attracting antibodies, such as primary macrophages and neutrophils, towards the location of the lesion. While macrophages are necessary for sweeping away debris and starting tissue healing, neutrophils aid in the removal of germs and foreign particles.

Chitosan and its byproducts accelerate wound healing by improving the abilities of cells associated with inflammation such as macrophages, polymorphonuclear leukocytes (PMN), osteoblasts, and fibroblasts [36]. Chitosan possesses qualities that make it an appealing component for biomedical applications and may serve to eliminate bacteria from the area of injury through the process of inflammation stage, among other applications. The anti-inflammatory characteristics of chitosan assist in modifying the immune system's response to inflammation by decreasing the production of chemokines and cytokines that are pro-inflammatory [37]. Chitosan has a range of immunomodulatory effects since it can produce a variety of cytokines. Additionally, it helps to lessen the accumulation of fluid and edema at the site of the wound.

It has been discovered that chitosan aids in the movement of inflammation-related cells that are necessary for the production of development factors and pro-inflammatory substances during the initial stages of the healing process. This is also capable of entering the nucleus of microorganisms, which is where it binds to the DNA as well as prevents the creation of mRNA and proteins [38].

Proliferative Phase

The initial inflammatory phase is followed by the proliferative phase. During the proliferation phase, various cellular processes are involved in repairing the damaged tissue.

The cell proliferation phase involves three phases, (1) neo-angiogenesis, (2) formulation of granulation tissue, and (3) ECM re-epithelialization.

Chitosan has the ability to stimulate platelet-derived growth factor (PDGF) and transforming growth factor (TGF) release. Chitosan supplies a non-protein matrix for the development of 3D tissues and stimulates macrophages to engage in tumor-curing functions [39]. Chitosan can act as a carrier for growth factors or cytokines that encourage cell movement, growth, as well as differentiation and also stimulate the construction of new vessels in the bloodstream (angiogenesis), that provide nutrients and oxygen for proliferating cells [40].

Skin Remodeling

Skin remodeling is a continual and dynamic process that happens throughout life. It refers to the natural regeneration and preservation of the framework and operations of the skin.

Chitosan precipitation across the dermis might help injured cells heal by forming a system that connects cells and stimulates collagen production while preserving adequate oxygen penetration. Additionally, this has strong biodegradability, biocompatibility, hemostatic, anti-inflammatory activities, antibacterial, beneficial exudate intake, and promotes rejuvenation of tissues as well as the formation of skin collagen fibers. It lends itself for use to be a component in a wide spectrum of dermatological treatments [41].

Due to their intriguing properties including biological suitability, biodegradable properties, and insufficient toxic effects that render them helpful to the medical area chitin and chitosan are now getting a lot of attention in their medical and pharmaceutical uses. Chitosan can be used as a covering for common biomedical materials and has an accelerating effect on wound healing. When chitosan is present, the promotion of adherence of platelets by plasma and extracellular matrix proteins has a considerable favorable impact on wound healing [42].

Biomaterials and clinical applications 

Medical Implants

Patients with joint conditions including osteoarthritis and rheumatoid arthritis could need surgical procedures like hip and knee replacements. Among the orthopedic implants utilized in these circumstances are appliances and parts for temporary fracture fixation, such as plates, screws, wires, and nails. Polymer biomaterials like polyethylene and polyetheretherketone (PEEK) and metals like titanium and stainless steel are used for implanting bone grafts [43].

Wound Healing

Biomaterials are used in the forms of scaffolds, hydrogels, impermeable films, alginates, hydrocolloids, and foams. Biomaterials are also employed in administering localized nucleic acids to chronic wounds that are non-healing. By focusing on gene pathways, they manipulate the process of wound healing toward a pro-healing environment. In Cold Atmospheric Pressure Plasma (CAP) therapy, biomaterials and temporary tissue restoration scaffolds are being used for their antibacterial effects, wound acidification, elevated microcirculation, and cell stimulation from a single application. Encouraging remodeling and epithelialization along these briefly implanted skin replacement scaffolds has the potential to promote wound healing. Recent developments include using 3D printing to dose topical formulations for wound dressings, using and controlling stem cells to create healing tools that mimic natural healing processes while also restoring factors like variables including cutaneous appendages, vascular plexus, and visceral tissues, as well as darkening [44,45].

Tissue Engineering

Biomaterials are crucial for growing tissues and organs in the lab for transplantation. They act as a scaffold on which cells can bind, and differentiate. A carefully crafted mixture of cells, spatiotemporally delivered biologically active substances and a customized scaffold framework are required for the engineering of complex tissues [46]. The direct implantation of a scaffold that has been specifically sized and shaped and is pre-fabricated from biocompatible biomaterials. By encouraging the host's tissue regeneration, it draws in nearby cells. As a result, it offers an immune-compatible material, preventing the resistance of the transplanted scaffold [47]. The capacity of tissue engineering biomaterials to promote cellular activity, molecular communication, and the required mechanical support in order to accelerate tissue regeneration is referred to as biocompatibility [48]. 

Biosensors

Biomaterials have been widely exploited to create effective biosensing prototypes, such as polymeric fibers and polymer composites combined with conducting materials.

To create more effective diagnostic systems, multidisciplinary design methods are used while creating biosensors employing different biomaterials. Natural biopolymeric materials like chitosan are used for developing biosensors made via protein immobilization [49].

*Pharmaceutical Applications*

Different delivery systems have been designed to lessen the persistent impacts of medications or elements like steroids when used in oral health issues like dental calculus, osseointegration, peri-implantitis, recovery, resorption of bone, and other associated diseases. The biomaterials consisting of organic polymeric substances such as gelatin, chitosan, hyaluronic acids, and calcium phosphate as well as metal-based transmitters like gold, titanium, and silver help to acquire the best bio-compatible compositions, control the flow of drug absorption, lower oral microbial ailments and reduce the dosing regularity. These technologies and equipment suggest efficient treatment delivery methods for oral disorders [50].

Biomaterials are employed in drug delivery by delivering cells and bioactive chemicals together with the deployment of a scaffold or support structure. They might be capable of migrating cells, growth-promoting agents, and water-soluble medications to a tissue defect in a way that fosters conditions for lasting cell sustenance, proliferation, and differentiation [51].

Utilizing biomaterials rather than non-biodegradable materials helps to regulate the pace of drug absorption, access optimal biocompatible compositions, and decrease the frequency of dosage.

Biomaterials are used in the manufacturing of silicone hydrogel contact lenses, rigid gas permeable contact lenses, soft hydrogel contact lenses, intraocular lens materials, contact lens care solutions, and cleaners, topical/intraocular anesthesia, and intraocular controlled drug delivery [52]. The well-known silicone hydrogel and hydroxy ethyl methacrylate (HEMA)-based lens materials have developed into contact lens (CL) materials. Due to the nature of these biomaterials, which are exceptionally biocompatible, cellulose, polyethylene glycol (PEG), chitosan, hyaluronic acid (HA), and other bioavailable compounds were transplanted or incorporated into these hydrogels [53].

Biomaterials are used as films in hair beauty products and transfer-resistant colors for cosmetics. They are also used as thickening agents and rheology agents in emulsions, hair colorants, and gels as well as moisturizers and conditioners. In order to protect hair and conform it to the desired styles, polymers used for hair conditioning and styling form films [54].

Several hydrogels that can be used as a "beauty mask" mostly contain collagen. This type of cosmetic will enhance anti-aging characteristics and restore skin elasticity [55].

Bioadhesive polymers provide adaptability to healthcare and pharmaceutical innovations. Incorporating these materials into typical forms of administration or surgical instruments could increase or enhance the adhesivity of the bioadhesive structures, extending their retention period at the point of intake or activity and resulting in a long-lasting absorption of active ingredients along with superior accessibility and medicinal benefits [56]. Using photochemical tissue bonding (PTB) has great potential in tissue repair as a suture-less approach. Rose Bengal (RB) combined with a chitosan film can be used as a bioadhesive, a substitute for sutures in nerve-repairing devices. When compared to suture repair, RB-chitosan adhesive was proven to lead to a speedier and more pronounced recovery of grip force [57].

Biomaterials have diverse properties including biocompatibility and remarkable physical characteristics, making them invaluable not only in these fields but also in the various fields of applications like surgery, biocompatible lubricants, bio-imaging, diagnostic tools, drug testing, organ regeneration/artificial organ, and so on.

Chitosan as a biomaterial

A biomaterial made from chitin called chitosan is very adaptable. It has been utilized for systemic and local medication and vaccine distribution, and its biocompatibility, ability to degrade, and bioactivity serve as an intriguing substance over a range of uses in the form of biomaterial in healthcare and drug companies [58]. Chitosan has high biological compatibility and low harmful effects because of the inherent structural stability of glycosaminoglycan [59]. Chitosan is currently being studied as an intriguing polysaccharide resource [60]. Chitosan has been reported to be extremely biodegradable, owing to the fact that its molecular chains can be digested by either lysozyme or chitinase under physiological circumstances [61]. It is also well tolerated by living things, including people, making it appropriate for use in a variety of pharmacological in addition to therapeutic uses. It acts as a biopolymer with minimal harmful effects and can be broken down through enzymatic hydrolysis using the proteolytic enzyme lysozyme [62].

Chitosan's antibacterial action uses two different methods. The first process is that the chitosan's positive charges may interact with the bacterial surface's negative charges, altering permeability and allowing solutes to escape the cells. The second process involves the possibility of binding with bacterial DNA cells, which prevents the manufacture of RNA [63].

Chitosan is a polycation because of its protonated amino groups, which may create ionized structures in a wide range of organic and synthetic anionic species, such as proteins, lipids, some negatively charged synthetic polymers, and DNA [64].

Chitosan is a versatile material that may be molded into a variety of objects, including sponges, films, gel, fibers, beads, and solutions and due to its adaptability, it can be customized for a variety of uses, including tissue engineering, drug delivery and wound healing.

**Figure 3:**
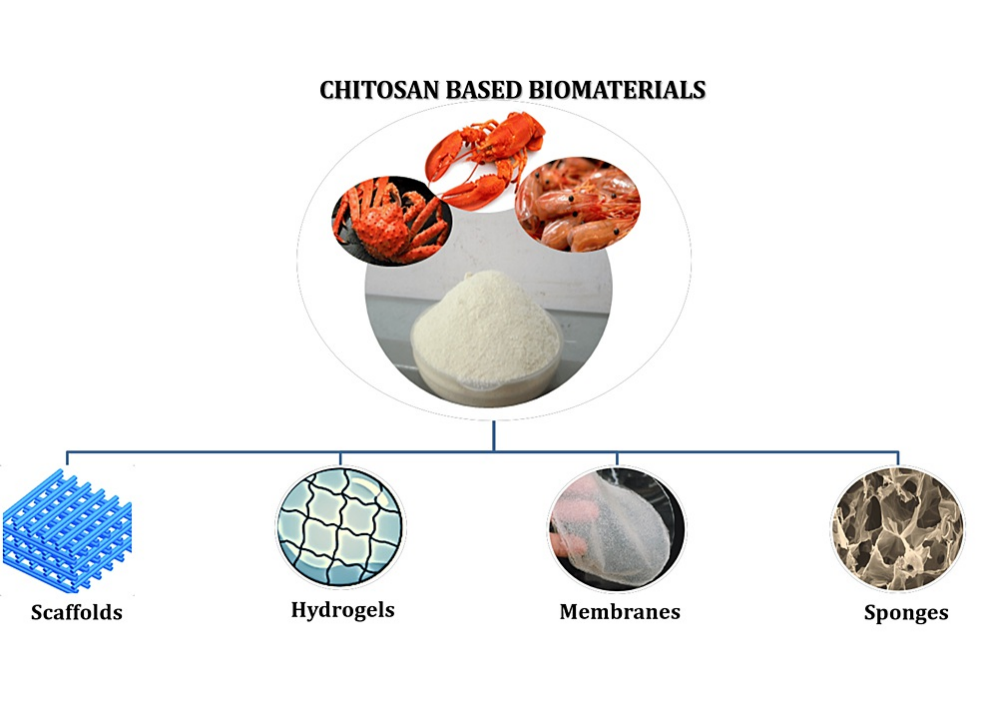
A schematic representation of the types of chitosan-based biomaterials

Chitosan could be used in various forms as a biomaterial which includes scaffolds, hydrogels, membranes, and sponges. A schematic representation of types of chitosan-based biomaterials is illustrated in Figure 3.

Overall, chitosan is a significant biomaterial with an array of applications in medical treatment, healthcare, cosmetics, and numerous industries attributable to its special mix of biocompatibility, biodegradability, antibacterial characteristics, and diversity in formulation. The chitosan-pectin-TiO2 covering material maintained an optimal level of control over the vaporized transpiration of water from wound beds, absorbed more exudates, and kept the wound beds moist without running the danger of exudate accumulation or dehydration [65].

Types of chitosan-based biomaterial applications

Chitosan-Based Fibrous Scaffold 

Chitosan/polycaprolactone scaffolds loaded with mupirocin and containing lidocaine hydrochloride (LID) are versatile wound dressings. The scaffolds were given a nanofibrous framework that improved the interface between the scaffold and cells in the plasma and demonstrated effective blood coagulation ability. Particularly, these scaffolds showed quick LID release and sustained mupirocin discharge. Mupirocin-containing CSLD-PCLM scaffold (mupirocin-loaded chitosan/polycaprolactone) had exceptional antibacterial efficacy. Additionally, in a full-thickness skin defect framework, the accumulation of collagen and complete re-epithelialization were both greatly facilitated by the scaffold during the wound healing process. As a result, these polymeric nanofibrous scaffolds are potential options for wound dressings in upcoming clinical applications and may perfectly suit the varied criteria of the wound healing process [66].

An electrically stimulated fluid of solutions of polymers is created on a spinning technique during electrospinning, employing the forces of electrostatic from the polymer solution to generate nanofibers. Chitosan has demonstrated an ideal result for applications involving the rejuvenation of tissues when electrospun with other bio-polymers (gelatin, chitosan, etc.) [67].

Nanofibers made of chitosan showed great promise for treating wounds. Their structure and makeup closely mimic the extracellular matrix found in the body, which promotes the regeneration of tissues. It is anticipated that chitosan's slow biodegradation will encourage its elimination, thus avoiding a need for tragic debridement. Furthermore, natural chitosan metabolites produced during biodegradation ought to encourage hepatic regeneration [68].

Nanofibers made of chitosan promote angiogenesis, cell migration, adhesion, and proliferation. Some fibers' hydrophilicity and bioactivity were increased by covering them with chitosan to accelerate wound healing. The fibrous membranes were shown to be biocompatible in in-vitro experiments using fibroblasts because they had good cell viability which enhanced cellular adhesion and growth. In actuality, only fibers coated in chitosan produced fully colonized tissues. Chitosan is useful for enhancing the characteristics of polylactic acid (PLA) nanofibers and creating composite fibrous membranes with potential uses in wound treatment [69].

Chitosan/polyvinyl alcohol (CS/PVA) fibrous membranes along with the antimicrobial enzyme lysozyme prevent wound infections [70]. When integrated into electro-spun chitosan/polyethylene oxide (CS/PEO) fibers, nano-silver particles demonstrated antibacterial activity that is linked to wound infections. Electro-spun CS/arginine fibers had faster wound healing and antibacterial characteristics [71].

As a wound treatment, carboxymethyl-hexanoyl chitosan nanofibers demonstrated good biocompatibility for fibroblasts while maintaining their antibacterial properties.

For third-degree burns, chitosan-nanofiber mats were developed and evaluated as an injury dressing. Chitosan-nanofiber coatings offered efficient exudate absorption, wound ventilation, infection prevention, and encouragement of the regrowth of skin tissue. The removal of these polymers minimized the amount of physical injury done to the lesion.

Leishmanial ulcers treated with CS/PEO nanofibers containing berberine (BBR) experienced quick macroscopic and microscopic wound healing, and the wound diameter was dramatically reduced. The greatest influence on the healing process came from chitosan scaffolds, which reduced the amount of wound tightening and promoted the growth of neo-dermis and re-epithelialization of the wound [72].

Chitosan nanofibers and PVA produced a scaffold with antibacterial properties. The fibroblast culture demonstrated that the generated nano-scaffold was ideal for the proliferation of fibroblast. Keratinocytes swelled, causing aberrant epidermis formation. If chitosan were added to the scaffold, healing would be enhanced, and the color of the wound site [73]. The various properties of chitosan-incorporated substances are demonstrated in Table 1.

**Table 1:** Demonstration of various properties of chitosan-based fibers

Chitosan	Substance incorporated	Properties
	Polylactic acid (PLA)	Cellular adhesion and proliferation, development of fibrous membrane
	Chitosan/polycaprolactone (CSLD-PCLM) + mupirocin with lidocaine hydrochloride (LID)	Blood coagulation, antibacterial activity, facilitates collagen deposition, sustained release of mupirocin, quick release of LID, and complete re-epithelialization
	Polyvinyl alcohol (PVA)	Antibacterial activity, fibroblast and keratinocyte proliferation, lighten the color of the wound site
	Polyethylene oxide (PEO) + berberine (BBR)	Reduced wound contraction, promotes the growth of the neodermis and re-epithelialization of the wound
	Carboxymethyl-hexanoyl	Biocompatibility for fibroblasts, antibacterial activity
	Arginine	Faster wound healing, antibacterial activity
	Polyvinyl alcohol (PVA) + lysozyme	Prevents infection of wounds
	Polyethylene oxide (PEO)	Enhanced antibacterial activity

Chitosan scaffolds offer active areas of research and innovation in wound healing as they hold the capacity for enhancing clinical results, reducing complications as well as improving the overall healing process. However refining scaffold design, exploring new drug delivery strategies, and conducting large-scale clinical trials are needed.

Chitosan Hydrogels

Chitosan is regarded as the perfect substance for hydrogels because of its ability to degrade, biological compatibility, non-toxicity, and antimicrobial properties. Characteristics, biological properties, hemostatic action, and capacity to easily react with other chemicals and undergo chemical change. Hydrogels are an important branch of chitosan material application since they have demonstrated potential as bone fracture fixation devices and wound dressings [74]. The hydrogels' porous structure allowed for a more gradual removal by the integrated antimicrobial chemical within the hydrogel matrices. According to Fasiku et al., the hydrogel proved to have a harmless effect and an excellent viscosity suitable for topical application [75]. Chitosan-based hydrogel dressings will have a higher capacity to hasten wound healing when modified and/or coupled with other polymers. Certain chitosan hydrogels can carry biologically active compounds (drugs, cultivated materials, embryonic cells) and control the discharge of the injury, such as the chitosan-PVA hydrogel [76]. Chitosan-based hydrogel's ability to maintain a moist wound environment was successful in stimulating tissue regeneration and epithelialization [77]. Chitosan-based hydrogel wound dressings have the potential to dramatically enhance wound repair when compared to inert hydrogels because of the polysaccharide's innate biological properties [78].

Chitosan-based hydrogels' enhanced bioactivity enables them to hasten vascular development and the formation of collagen development enhances inflammatory cellular activity, and shields injuries via microorganisms, all crucial steps in the healing of wounds, especially those that are infected. Hydrogels created solely from chitosan and cross-linking agents, however, lack sufficient antibacterial characteristics due to their limited solubility [79,80]. Chitosan hydrogels may stimulate cell movement and growth, both of those being crucial processes during the healing of wounds. They may additionally increase the activity of keratinocytes, and vascular cells along with fibroblasts, as well as stimulate angiogenesis. A hydrogel may experience external mechanical force when inserted under the skin or employed as an external dressing on the injury site, thereby shortening the dressing's lifespan. The hydrogel's ability to mend itself allows it to keep its underlying structure, recover its primary functions, and extend the life of the dressing. The hydrogels that Craciun et al. experimented with showed good stability, microporous anatomy, and porosity width determined by the fluid content, and degradation in aquatic conditions regulated by pH [81].

The disc diffusion approach was employed to evaluate the hydrogel specimens for their ability to kill bacteria. The findings revealed that the chitosan-based hydrogels had effective antibacterial properties towards Gram-negative bacteria *Escherichia coli* strains which is explained by how chitosan interacts with the bacteria's plasma membrane [82]. Chitosan hydrogels' main uses in wound healing include their use as hemostatic agents and wound dressings to hasten the recovery process. The permeability of chitosan-based hydrogels possesses a significant impact on parameters like swelling, adherence of cells, and cell growth rate that are essential to tissue formation in scaffolds employed for tissue engineering. Self-healing hydrogels' injectability in tissue regeneration, personalized drug administration, and suitability as a material for 3D and 4D printing [83].

Chitosan Membranes

Chitosan membranes are a form of biomaterial used in wound healing that can speed up and enhance the healing process. On the basis of synthetic or natural polymers, arrays of wound-dressing products were created. There are several different primary categories of wound-dressing resources, including, gels, hydrocolloids, films, membranes, fibers, and sponges. A "membrane" can refer to an instrument meant to separate an area from its surroundings. The majority of the time, membranes are hydrate films used in biological and medical research. Research has demonstrated that cross-linking improves the durability and adsorption capability of chitosan membranes [84]. Chitosan is initially dissolved in a solution of acetic acid in the solution casting-evaporation method, considered to be an extremely fundamental technique for generating chitosan membranes. The primary drawback of this strategy is that mammalian cells are fatally affected by acetic acid and other chemical cross-linkers like glutaraldehyde and carbodiimide. As a result, the speed at which wounds can heal is significantly constrained by this strategy. Matica et al. produced chitosan-glycerol membranes packed alongside silver sulfadiazine (AgSD) along with tetracycline hydrochloride (TH) using an easy casting-evaporation technique, as well as they assessed the inhibition rate of bacterial proliferation against *Escherichia coli* and *Staphylococcus aureus *through determining the inhibition region diameter [85].

Azad et al. observed that keratinocyte generation was more common and re-epithelialization moved forward more swiftly in the regions coated with chitosan mesh membrane than in the vehicle for measurement [86]. Upon completing the process of re-epithelialization, there had been no dead tissue in the chitosan-dressed region [86]. The application of chitosan-alginate membrane for the cure of ulcerated skin is also having an excellent effect on every stage of the healing process. Chitosan-alginate membrane was found to help regulate all phases of wound healing [86]. 

Chitosan membranes can be made to fit a variety of sizes and types of wounds. They are utilized in an array of medical contexts, like surgical scar dressings, burn treatment, and the handling of long-term injuries. The efficiency of chitosan membranes can, however, vary based on the kind of wound, the patient, as well as the specific chitosan formulation getting utilized.

Chitosan Sponges

It is envisaged that controlling the arrangement of pores and functional remodeling will improve the anti-infective and hemostatic properties of the hemostats. A simple and manageable architectural feature that can facilitate the transfer of air, nourishments, and metabolic products as well as blood circulation, cell host permeation, and interaction with the tissues around it is the incorporation of microchannels into three-dimensional (3D) structures. Hemostats have been made using chitosan because of its unique qualities. As a result of the potent water-resistant exchanges that exist among alkyl chains and the membranes of red blood cells (RBCs), microbes, and platelets, placing water-repellent alkyl chains inside a chitosan core might enhance its hemostatic and anti-infective properties. A microchannel structure that has been integrated into a chitosan sponge allows for quick shape recovery through the absorption of blood and water. This chitosan-based sponge has greater pro-coagulant capacity under laboratory conditions and hemostatic ability on models of perforation, heparinization, and lethally normal wounds. This demonstrates their ability to effectively treat potentially fatal noncompressible hemorrhages and promote wound healing on a clinically significant level [87].

Due to the sponge's high porosity nature, controlling the medication release from it can be fairly challenging. High structural porosity and the hydrophilic nature of chitosan cause quick dissolution of any activated compound, particularly when using the water-based ingredient. Thus, chitosan could be modified to act as a controlled release system by minimizing its hydrophilicity [88].

Blood moisture may be absorbed by hydroxy-butyl chitosan and chitosan, an integrated sponge that has excellent hydrophilicity, which can boost the concentration of blood and consistency and produce a semi-swelling fluid colloidal material that can block blood vessels. The integrated sponge has no harmful effects on cells as well as can encourage fibroblast development. The capacity of the epithelial cells to adhere to the integrated sponge and penetrate its inner layers to facilitate wound healing, aid in the quicker development of skin glands, and promote re-epithelialization encourages the application of such sponge for dressings for injuries [89]. The use of chitosan sponges that have been treated with antibiotics has increased lately. There are still few clinical investigations on the use of chitosan sponges to deliver topical antibiotics. Future research on the association between alcohol consumption and wound dehiscence and dosage response should be considered [90].

Chitosan biomaterials in therapeutics

**Figure 4:**
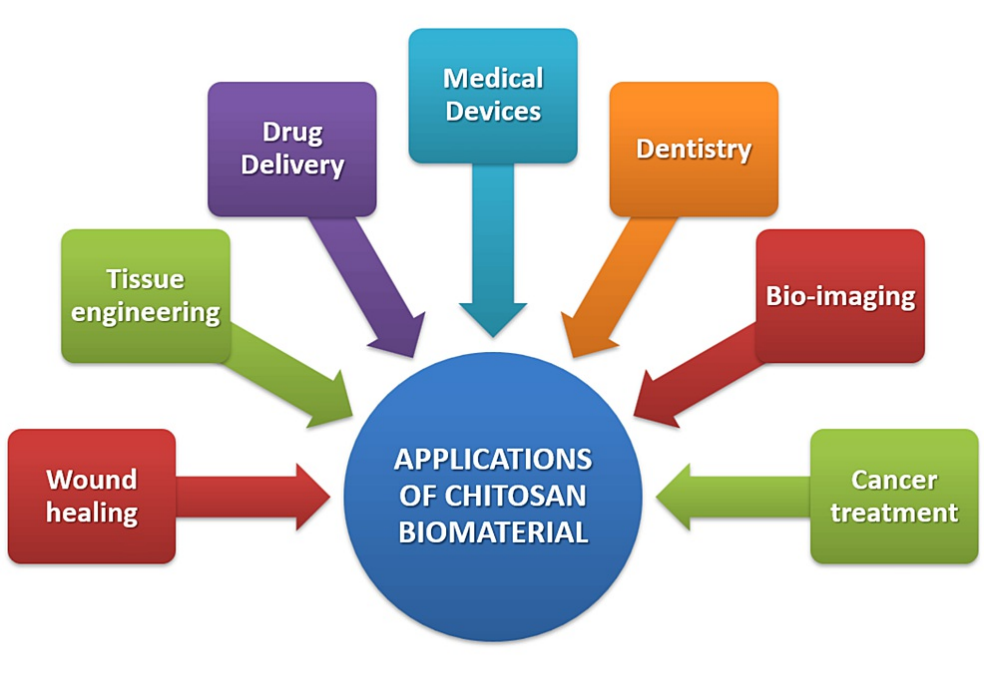
Various applications of chitosan-based biomaterials

Chitosan-based materials, because of their bio-compatibility and ability to function as sites of interaction alongside biologically active substances, have been widely explored in the oral route for the delivery of medications as well as methods for topical distribution, gene delivery, and even cancer therapy. Various applications of chitosan-based biomaterials are illustrated in Figure 4.

Diabetic Wound Healing

Chitosan biomaterials have shown potential in diabetic wound healing, where the healing process can be hampered by diabetes-related factors such as reduced blood circulation and compromised immunological function. Mirbagheri et al. discovered that electrospun chitosan nanofibres offer a promising therapy option for controlling diabetes and chronic wounds [91]. The use of chitosan films can have a direct impact on the cost of diabetic foot therapy and amputation surgery [92].

Chitosan hydrogels are being found to enhance the healing of wounds in diabetic wound ulcers by offering a moist atmosphere to the injuries, facilitating cell migration and tissue regeneration [93]. Chitosan gels and films were reported to hinder Gram-positive and Gram-negative bacteria emergence in people with diabetes with ulcerated skin [94].

Burns

Chitosan nanofibers demonstrated fibroblast proliferation and angiogenesis in second-degree burned areas in rats, while chitosan coverings cured second-degree burns in people in vivo trials. Chitosan hydrogels are being demonstrated to heal third-degree burns without causing irritation or injury [95]. Chitosan membranes act as a physical barrier over burn wounds, keeping external pollutants, debris, and infection at bay. In situ, injectable chitosan hydrogels show changes from inflammation to proliferative phase in full-thickness third-degree wounds from burns of Wistar albino rats [96].

Drug Delivery

As a viable local drug delivery system, ornidazole-loaded PVA/carboxymethyl-chitosan blend films were investigated. A small number of investigations in mammalian models have revealed that chitosan-derived delivery methods are safe and biocompatible. As a result, it is feasible to modulate drug release and improve solubility or absorption, hence increasing or maintaining pharmacodynamics and biological responses [97]. Chitosan nanoparticles, due to their smaller particle size, can facilitate controlled drug release, improving drug stability and bioavailability by penetrating the mucous layer [98].

Chitosan in Tissue Engineering

Tissue engineering is a well-studied topic in which chitosan is utilized to make polymeric scaffolds for the reconstruction and regeneration of tissues [99]. In tissue engineering, which aims to develop functional and biocompatible substitutes for injured or destroyed tissues and organs, chitosan membranes play a vital role, whereas chitosan hydrogels demonstrate effective bone regeneration. Chitosan film containing titanium dioxide nanoparticles has both mechanical and biological regeneration characteristics. The membrane build exhibited excellent physical, crystallization, and flexibility characteristics. Membranes also demonstrated antimicrobial effects towards Staphylococcus aureus [100]. Chitosan sponges may be employed in filler substances in tissue engineering of bones.

Dentistry

Antibiotics (such as metronidazole, chlorhexidine, and nystatin) can be delivered to oral cells in actual conditions to fight off fungal diseases and oral stomatitis using chitosan in the form of nanoparticles and resorbable films [101]. Interesting physical characteristics of chitosan gels may be influenced by chitosan concentration. In fact, it has been demonstrated that these chitosan-based gels (1-4%) possess intriguing consistency that allows them to be administered inside spaces in the periodontal tissue. Moreover, it could be trusted to deliver active medications to the site of the disease [102].

Here, chitosan-based restorative materials are being considered for effectively delivering organic amelogenin at the locations of enamel defects. The application of chitosan antioxidant gel to dentin increases the bonding of composite resins while also increasing impermeability. According to some studies, chitosan hydrogel can produce a strong dentin bonding system and boost shear bond strength [103]. Numerous researches have demonstrated that experimental chitosan-based biomaterials can enhance the preservation and neoformation of dental pulp tissue, indicating the possibility of using the polymer for pulp structure restoration and preservation [104].

Chitosan in Biosensors

Biosensors can be created using chitosan nanofibers because of their excellent biocompatibility and high enzyme loading capacity. The nanoparticle incorporation of gold in the network of composite chitosan nanofibers provides the biosensor's superior electrochemical performance in the detection of cholesterol [105].

Molecular Imaging

Chitosan and its derivatives offer an appealing toolbox for complex molecular imaging applications. Although there is undoubtedly still much to be done before chitosan's full potential can be realized, the advantages are substantial enough to justify the effort [106].

Bioprinting

Chitosan biomaterials are becoming more popular and are being used in the cutting-edge technology of bioprinting, which is used in stem cell therapy and tissue engineering. Complex 3D structures can be made by precisely depositing living cells and substances via bioprinting. Bioprinting is the process of assembling living and non-living components according to a specified 2D or 3D pattern using technologically assisted transmit technologies in order to create bio-engineered frameworks.

The process of putting bioprinting into practice entails several steps, which are referred to as (i) plan, (ii) print, and (iii) process. These steps indicate (1) designing the whole print layout and the bioprinting elements (such as bio-inks, cells, and biomaterial inks), (2) using the right bioprinter to print the desired construct, and (3) processing the generated construct. Finding the biological question of interest is the first step in the process; it will naturally direct other choices made during the planning and printing phases [107].

Chitosan in Bioprinting

Chitosan is a strong option in wound healing and tissue bioprinting in 3D due to its hydrophilic nature, which encourages nearly every form of cell by attachment and grows within the scaffolds [108]. In human mesenchymal stem cells (MSCs), cell differentiation into osteogenic or adipogenic lineage has been affected by bio-printed agarose, collagen, chitosan-agarose, and collagen-agarose hydrogels [109].

The printed bio-inks based on chitosan have special properties like outstanding cell/matrix encounters, replicating the structure of original tissue, offering an atmosphere for nutrition and oxygen transfers, and generating a favorable immunological response after implantation. Chitosan may have strong antibacterial properties when present in bio-ink formulations. Bacterial mortality or growth restriction results from the capacity of protonated NH3+ in chitosan to interact electrostatically and penetrate through the negative shell membrane of bacteria.

When used in bone, vascular, and cartilage tissue, extrusion-based and laser-based bioprinting of chitosan demonstrated properties like durability under physiological circumstances, a relevant variety of the solution viscosity numbers, usefulness, survival of cells, growth, and division, and printing capability [110]. Skin, cartilage, bone, blood vessels, and other tissues and organ constructions can be made using chitosan-based bioprinting. These concepts might be used in transplantation and regenerative medicine.

The printed hydrogel constructions remained mechanically stable under physiological settings, and the polyelectrolyte chitosan gelatin hydrogels also showed excellent biocompatibility with human skin cells and high printability [111]. Due to their excellent biocompatibility, chemical gel formation ability, moderate breakdown, and ECM element modeling properties, chitosan additives have become widely used as cell-laden "bio-inks" for liver cells and 3D bioprinting of organs. Gelatin was added to the chitosan to modify it, resulting in printable polyelectrolyte gelatin-chitosan (PGC) hydrogels. The PGC hydrogels have been optimized for bioprinting skin structures. The viscosity of the PGC hydrogels was sufficiently high. A chitosan solution was used to 3D print polyethylene terephthalate. Chlorhexidine was infused into each coating of the polyethylene terephthalate-chitosan cloth, a heating device, which also increased the integrity of the porous 3D-printed scaffold and increased the distribution period of chlorhexidine. Chitosan can be co-printed into irregular structures with other natural polymers, and typically, the lowermost section is capable of touching the skin that is injured [111,112].

For medication testing and illness research, in vitro disease models made from bio-printed tissues utilizing chitosan-based bio-inks can be used. In order to create dental implants, oral mucosa, and periodontal tissues, they are also employed in bioprinting for dental and oral tissue engineering. Bioprinting will revolutionize healthcare in the coming years by making it possible to create functional, patient-specific tissues and organs and by fostering the production of cutting-edge medicinal solutions. The potential for bioprinting applications is numerous and offers great promise for enhancing the quality of life and healthcare outcomes as research and technology advance.

## Conclusions

In summary, the utilization of chitosan-based biomaterials in wound healing presents both promise and challenges. These natural polymers have shown significant potential in promoting wound closure, tissue regeneration, and infection control. The biocompatibility and biodegradability of chitosan indicate that it is a desirable choice for surgical dressings as well as scaffolds, with minimal adverse effects reported. However, critical assessment reveals several areas that warrant further investigation. The effectiveness of chitosan-based materials may depend on variables like origin, molecular weight, and processing techniques. Achieving consistent results and optimizing material properties remain ongoing challenges.

Moreover, while chitosan's antimicrobial properties are advantageous, their long-term impact on the wound microbiome needs careful consideration. Additionally, more comprehensive clinical studies are necessary to establish the safety and efficacy of chitosan-based items that treat wounds. In conclusion, chitosan-based biomaterials has demonstrated its ability in healing of wounds, but a deeper understanding of their properties, standardization in manufacturing, and rigorous clinical validation are crucial steps to harness their full therapeutic potential and ensure their safe and effective use in clinical practice.

## References

[1] Oleksy M, Dynarowicz K, Aebisher D (2023). Advances in biodegradable polymers and biomaterials for medical applications-a review. Molecules.

[2] Elangwe CN, Morozkina SN, Olekhnovich RO, Krasichkov A, Polyakova VO, Uspenskaya MV (2022). A review on chitosan and cellulose hydrogels for wound dressings. Polymers (Basel).

[3] Lungu R, Paun MA, Peptanariu D (2022). Biocompatible chitosan-based hydrogels for bioabsorbable wound dressings. Gels.

[4] Biomaterials. https://www.materialstoday.com/biomaterials/journals/biomaterials/.

[5] Pires PC, Mascarenhas-Melo F, Pedrosa K (2023). Polymer-based biomaterials for pharmaceutical and biomedical applications: a focus on topical drug administration. Eur Polym J.

[6] (2017). What are biomaterials?. https://www.nibib.nih.gov/science-education/science-topics/biomaterials.

[7] Baranwal J, Barse B, Fais A, Delogu GL, Kumar A (2022). Biopolymer: a sustainable material for food and medical applications. Polymers (Basel).

[8] Henao J, Poblano-Salas CA, Monsalve M (2019). Bio-active glass coatings manufactured by thermal spray: a status report. J Mater Res Technol.

[9] Punj SSK, Singh J, Singh K (2021). Ceramic biomaterials: properties, state of the art and future prospectives. Ceram Int.

[10] Kohane DS, Langer R (2008). Polymeric biomaterials in tissue engineering. Pediatr Res.

[11] Teo AJ, Mishra A, Park I, Kim YJ, Park WT, Yoon YJ (2016). Polymeric biomaterials for medical implants and devices. ACS Biomater Sci Eng.

[12] Abourehab MA, Rajendran RR, Singh A (2022). Alginate as a promising biopolymer in drug delivery and wound healing: a review of the state-of-the-art. Int J Mol Sci.

[13] Kalirajan C, Dukle A, Nathanael AJ, Oh TH, Manivasagam G (2021). A critical review on polymeric biomaterials for biomedical applications. Polymers (Basel).

[14] de Sousa Victor R, Marcelo da Cunha Santos A, Viana de Sousa B, de Araújo Neves G, Navarro de Lima Santana L, Rodrigues Menezes R (2020). A review on chitosan's uses as biomaterial: tissue engineering, drug delivery systems and cancer treatment. Materials (Basel).

[15] Ahmed S, Ikram S (2016). Chitosan based scaffolds and their applications in wound healing. Ach Life Sci.

[16] Wang W, Meng Q, Li Q, Liu J, Zhou M, Jin Z, Zhao K (2020). Chitosan derivatives and their application in biomedicine. Int J Mol Sci.

[17] Hu B, Guo Y, Li H, Liu X, Fu Y, Ding F (2021). Recent advances in chitosan-based layer-by-layer biomaterials and their biomedical applications. Carbohydr Polym.

[18] Abourehab MA, Pramanik S, Abdelgawad MA, Abualsoud BM, Kadi A, Ansari MJ, Deepak A (2022). Recent advances of chitosan formulations in biomedical applications. Int J Mol Sci.

[19] Luo Y, Wang Q (2013). Recent advances of chitosan and its derivatives for novel applications in food science. J Food Process Beverages.

[20] Singh R, Shitiz K, Singh A (2017). Chitin and chitosan: biopolymers for wound management. Int Wound J.

[21] Jafari H, Bernaerts KV, Dodi G, Shavandi A (2020). Chitooligosaccharides for wound healing biomaterials engineering. Mater Sci Eng C Mater Biol Appl.

[22] Zarei F, Soleimaninejad M (2018). Role of growth factors and biomaterials in wound healing. Artif Cells Nanomed Biotechnol.

[23] Wang CH, Cherng JH, Liu CC (2021). Procoagulant and antimicrobial effects of chitosan in wound healing. Int J Mol Sci.

[24] Cheung RC, Ng TB, Wong JH, Chan WY (2015). Chitosan: an update on potential biomedical and pharmaceutical applications. Mar Drugs.

[25] Buzlama A, Doba S, Alexey S (2020). Pharmacological and biological effects of chitosan. Res J Pharm Technol.

[26] Xia W, Liu P, Zhang J (2011). Biological activities of chitosan and chitooligosaccharides. Food Hydrocolloids.

[27] Ahmadi F, Oveisi Z, Samani SM, Amoozgar Z (2015). Chitosan based hydrogels: characteristics and pharmaceutical applications. Res Pharm Sci.

[28] Kim SY (2018). Competitive biological activities of chitosan and its derivatives: antimicrobial, antioxidant, anticancer, and anti-inflammatory activities. Int J Polym Sci.

[29] Park JK, Chung MJ, Choi HN, Park YI (2011). Effects of the molecular weight and the degree of deacetylation of chitosan oligosaccharides on antitumor activity. Int J Mol Sci.

[30] Broughton G 2nd, Janis JE, Attinger CE (2006). Wound healing: an overview. Plast Reconstr Surg.

[31] Cañedo-Dorantes L, Cañedo-Ayala M (2019). Skin acute wound healing: a comprehensive review. Int J Inflam.

[32] Landén NX, Li D, Ståhle M (2016). Transition from inflammation to proliferation: a critical step during wound healing. Cell Mol Life Sci.

[33] Xue M, Jackson CJ (2015). Extracellular matrix reorganization during wound healing and its impact on abnormal scarring. Adv Wound Care (New Rochelle).

[34] Feng P, Luo Y, Ke C (2021). Chitosan-based functional materials for skin wound repair: mechanisms and applications. Front Bioeng Biotechnol.

[35] Wang YH, Liu CC, Cherng JH (2019). Evaluation of chitosan-based dressings in a swine model of artery-injury-related shock. Sci Rep.

[36] Dai T, Tanaka M, Huang YY, Hamblin MR (2011). Chitosan preparations for wounds and burns: antimicrobial and wound-healing effects. Expert Rev Anti Infect Ther.

[37] Fong D, Hoemann CD (2018). Chitosan immunomodulatory properties: perspectives on the impact of structural properties and dosage. Future Sci OA.

[38] Zhang MX, Zhao WY, Fang QQ (2021). Effects of chitosan-collagen dressing on wound healing in vitro and in vivo assays. J Appl Biomater Funct Mater.

[39] Liu H, Wang C, Li C (2018). A functional chitosan-based hydrogel as a wound dressing and drug delivery system in the treatment of wound healing. RSC Adv.

[40] Zhang M, An H, Zhang F (2023). Prospects of using chitosan-based biopolymers in the treatment of peripheral nerve injuries. Int J Mol Sci.

[41] Guzmán E, Ortega F, Rubio RG (2022). Chitosan: a promising multifunctional cosmetic ingredient for skin and hair care. Cosmetics.

[42] Al-Rooqi MM, Hassan MM, Moussa Z (2022). Advancement of chitin and chitosan as promising biomaterials. J Saudi Chem Soc.

[43] Al-Shalawi FD, Mohamed Ariff AH, Jung DW, Mohd Ariffin MK, Seng Kim CL, Brabazon D, Al-Osaimi MO (2023). Biomaterials as implants in the orthopedic field for regenerative medicine: metal versus synthetic polymers. Polymers (Basel).

[44] Niculescu AG, Grumezescu AM (2022). An up-to-date review of biomaterials application in wound management. Polymers (Basel).

[45] Berger AG, Chou JJ, Hammond PT (2021). Approaches to modulate the chronic wound environment using localized nucleic acid delivery. Adv Wound Care (New Rochelle).

[46] Kretlow JD, Young S, Klouda L, Wong M, Mikos AG (2009). Injectable biomaterials for regenerating complex craniofacial tissues. Adv Mater.

[47] Eldeeb AE, Salah S, Elkasabgy NA (2022). Biomaterials for tissue engineering applications and current updates in the field: a comprehensive review. AAPS PharmSciTech.

[48] Ibrahim M, El-Wassefy NA, Farahat D (2017). 8 - Biocompatibility of dental biomaterials. Biomater Oral Dent Tissue Eng.

[49] Prasad A, Mahato K, Maurya PK, Chandra P (2016). Biomaterials for biosensing applications. J Anal Bioanal Tech.

[50] Hakim LK, Yazdanian M, Alam M (2021). Biocompatible and biomaterials application in drug delivery system in oral cavity. Evid Based Complement Alternat Med.

[51] Kim JJ, Evans GR (2012). Applications of biomaterials in plastic surgery. Clin Plast Surg.

[52] Salamone JC, Salamone AB, Swindle-Reilly K, Leung KX, McMahon RE (2016). Grand challenge in biomaterials-wound healing. Regen Biomater.

[53] Musgrave CS, Fang F (2019). Contact lens materials: a materials science perspective. Materials (Basel).

[54] Sionkowska A, Lewandowska K, Płanecka A, Szarszewska P, Krasinska K, Kaczmarek B, Kozlowska J (2013). Biopolymer blends as potential biomaterials and cosmetic materials. Key Eng Mater.

[55] Sionkowska A, Adamiak K, Musiał K, Gadomska M (2020). Collagen based materials in cosmetic applications: a review. Materials (Basel).

[56] Ahmady A, Abu Samah NH (2021). A review: Gelatine as a bioadhesive material for medical and pharmaceutical applications. Int J Pharm.

[57] Azuma K, Izumi R, Osaki T (2015). Chitin, chitosan, and its derivatives for wound healing: old and new materials. J Funct Biomater.

[58] Senel S, McClure SJ (2004). Potential applications of chitosan in veterinary medicine. Adv Drug Deliv Rev.

[59] Jafernik K, Ładniak A, Blicharska E, Czarnek K, Ekiert H, Wiącek AE, Szopa A (2023). Chitosan-based nanoparticles as effective drug delivery systems-a review. Molecules.

[60] Kumar MN (2000). A review of chitin and chitosan applications. React Func Polym.

[61] Guarnieri A, Triunfo M, Scieuzo C (2022). Antimicrobial properties of chitosan from different developmental stages of the bioconverter insect Hermetia illucens. Sci Rep.

[62] Jiménez-Gómez CP, Cecilia JA (2020). Chitosan: a natural biopolymer with a wide and varied range of applications. Molecules.

[63] Ibrahim HM, Zairy EM (2015). Chitosan as a biomaterial — structure, properties, and electrospun nanofibers. In InTech eBooks. Concepts, compounds and the alternatives of antibacterials.

[64] Croisier F, Jérôme C (2013). Chitosan-based biomaterials for tissue engineering. Eur Polym J.

[65] Deepachitra R, Lakshmi RP, Sivaranjani K, Chandra JH, Sastry TP (2015). Nanoparticles embedded biomaterials in wound treatment: a review. J Chem Pharm Sci.

[66] Yang S, Li X, Liu P, Zhang M, Wang C, Zhang B (2020). Multifunctional chitosan/polycaprolactone nanofiber scaffolds with varied dual-drug release for wound-healing applications. ACS Biomater Sci Eng.

[67] Qasim SB, Zafar MS, Najeeb S, Khurshid Z, Shah AH, Husain S, Rehman IU (2018). Electrospinning of chitosan-based solutions for tissue engineering and regenerative medicine. Int J Mol Sci.

[68] Lungu R, Anisiei A, Roşca I, Sandu AI, Ailincai D, Marin L (2021). Double functionalization of chitosan based nanofibers towards biomaterials for wound healing. React Func Polym.

[69] Milanesi G, Vigani B, Rossi S, Sandri G, Mele E (2021). Chitosan-coated poly(lactic acid) nanofibres loaded with essential oils for wound healing. Polymers (Basel).

[70] Charernsriwilaiwat N, Opanasopit P, Rojanarata T, Ngawhirunpat T (2012). Lysozyme-loaded, electrospun chitosan-based nanofiber mats for wound healing. Int J Pharm.

[71] Zhou Y, Yang D, Chen X, Xu Q, Lu F, Nie J (2008). Electrospun water-soluble carboxyethyl chitosan/poly(vinyl alcohol) nanofibrous membrane as potential wound dressing for skin regeneration. Biomacromolecules.

[72] Seyyed Tabaei SJ, Rahimi M, Akbaribazm M, Ziai SA, Sadri M, Shahrokhi SR, Rezaei MS (2020). Chitosan-based nano-scaffolds as antileishmanial wound dressing in BALB/c mice treatment: characterization and design of tissue regeneration. Iran J Basic Med Sci.

[73] Gomes SR, Rodrigues G, Martins GG, Roberto MA, Mafra M, Henriques CM, Silva JC (2015). In vitro and in vivo evaluation of electrospun nanofibers of PCL, chitosan and gelatin: a comparative study. Mater Sci Eng C Mater Biol Appl.

[74] El-Kased RF, Amer RI, Attia D, Elmazar MM (2017). Honey-based hydrogel: In vitro and comparative In vivo evaluation for burn wound healing. Sci Rep.

[75] Fasiku VO, Omolo CA, Devnarain N (2021). Chitosan-based hydrogel for the dual delivery of antimicrobial agents against bacterial methicillin-resistant staphylococcus aureus biofilm-infected wounds. ACS Omega.

[76] Alnazza Alhamad A, Zeghoud S, Ben Amor I, Hemmami H (2023). Chitosan-based hydrogels for wound healing: correspondence. Int J Surg.

[77] Kibungu C Kondiah PP, Kumar P, Choonara YE (2021). This review recent advances in chitosan and alginate‐based hydrogels for wound healing application. Front Mater.

[78] Takei T, Nakahara H, Ijima H, Kawakami K (2012). Synthesis of a chitosan derivative soluble at neutral pH and gellable by freeze-thawing, and its application in wound care. Acta Biomater.

[79] Wang X, Song R, Johnson M (2021). An injectable chitosan-based self-healable hydrogel system as an antibacterial wound dressing. Materials (Basel).

[80] Wang X, Song R, Johnson M (2023). Chitosan-based hydrogels for infected wound treatment. Macromol Biosci.

[81] Craciun AM, Morariu S, Marin L (2022). Self-healing chitosan hydrogels: preparation and rheological characterization. Polymers (Basel).

[82] Alven S, Aderibigbe BA (2020). Chitosan and cellulose-based hydrogels for wound management. Int J Mol Sci.

[83] Pellá MC, Lima-Tenório MK, Tenório-Neto ET, Guilherme MR, Muniz EC, Rubira AF (2018). Chitosan-based hydrogels: From preparation to biomedical applications. Carbohydr Polym.

[84] Spoială A, Ilie CI, Dolete G (2022). Preparation and characterization of chitosan/TiO(2) composite membranes as adsorbent materials for water purification. Membranes (Basel).

[85] Matica MA, Aachmann FL, Tøndervik A, Sletta H, Ostafe V (2019). Chitosan as a wound dressing starting material: antimicrobial properties and mode of action. Int J Mol Sci.

[86] Azad AK, Sermsintham N, Chandrkrachang S, Stevens WF (2004). Chitosan membrane as a wound-healing dressing: characterization and clinical application. J Biomed Mater Res B Appl Biomater.

[87] Du X, Liu Y, Wang X (2019). Injectable hydrogel composed of hydrophobically modified chitosan/oxidized-dextran for wound healing. Mater Sci Eng C Mater Biol Appl.

[88] Phaechamud T, Yodkhum K, Charoenteeraboon J, Tabata Y (2015). Chitosan-aluminum monostearate composite sponge dressing containing asiaticoside for wound healing and angiogenesis promotion in chronic wound. Mater Sci Eng C Mater Biol Appl.

[89] Hu S, Bi S, Yan D, Zhou Z, Sun G, Cheng X, Chen X (2018). Preparation of composite hydroxybutyl chitosan sponge and its role in promoting wound healing. Carbohydr Polym.

[90] McKee K, Easton J, Mullis B, Hadad I (2023). Chitosan sponges are associated with higher rates of wound complications compared to calcium sulfate beads. Cureus.

[91] Mirbagheri MS, Akhavan-Mahdavi S, Hasan A, Kharazmi MS, Jafari SM (2023). Chitosan-based electrospun nanofibers for diabetic foot ulcer management; recent advances. Carbohydr Polym.

[92] Escárcega-Galaz AA, Cruz-Mercado JL, López-Cervantes J, Sánchez-Machado DI, Brito-Zurita OR, Ornelas-Aguirre JM (2018). Chitosan treatment for skin ulcers associated with diabetes. Saudi J Biol Sci.

[93] Ko A, Liao C (2023). Hydrogel wound dressings for diabetic foot ulcer treatment: status‐quo, challenges, and future perspectives. BMEMat.

[94] Xu Y, Hu Q, Wei Z (2023). Advanced polymer hydrogels that promote diabetic ulcer healing: mechanisms, classifications, and medical applications. Biomater Res.

[95] Sánchez‐Machado DI, López-Cervántes J, Martínez‐Ibarra DM, Escárcega-Galaz AA, Vega-Cázarez CA (2022). The use of chitosan as a skin-regeneration agent in burns injuries: a review. e-Polymers.

[96] Bai Q, Zheng C, Chen W (2022). Current challenges and future applications of antibacterial nanomaterials and chitosan hydrogel in burn wound healing. Mater Adv.

[97] Fonseca-Santos B, Chorilli M (2017). An overview of carboxymethyl derivatives of chitosan: their use as biomaterials and drug delivery systems. Mater Sci Eng C Mater Biol Appl.

[98] Li J, Cai C, Li J (2018). Chitosan-based nanomaterials for drug delivery. Molecules.

[99] Elieh-Ali-Komi D, Hamblin MR (2016). Chitin and chitosan: production and application of versatile biomedical nanomaterials. Int J Adv Res (Indore).

[100] Madni A, Kousar R, Naeem N, Wahid F (2021). Recent advancements in applications of chitosan-based biomaterials for skin tissue engineering. J Bioresour.

[101] Husain S, Al-Samadani KH, Najeeb S, Zafar MS, Khurshid Z, Zohaib S, Qasim SB (2017). Chitosan biomaterials for current and potential dental applications. Materials (Basel).

[102] Aguilar A, Zein N, Harmouch E (2019). Application of chitosan in bone and dental engineering. Molecules.

[103] Gayen K, Pabale S, Shirolkar S, Sarkar S, Roychowdhury S (2022). Chitosan biomaterials: natural resources for dentistry. Int J Oral Health Sci.

[104] Kim Y, Zharkinbekov Z, Raziyeva K (2023). Chitosan-based biomaterials for tissue regeneration. Pharmaceutics.

[105] Khan A, Alamry KA (2021). Recent advances of emerging green chitosan-based biomaterials with potential biomedical applications: A review. Carbohydr Res.

[106] Agrawal P, Strijkers GJ, Nicolay K (2010). Chitosan-based systems for molecular imaging. Adv Drug Deliv Rev.

[107] Daly AC, Prendergast ME, Hughes AJ, Burdick JA (2021). Bioprinting for the biologist. Cell.

[108] Li S, Tian X, Fan J, Tong H, Ao Q, Wang X (2019). Chitosans for tissue repair and organ three-dimensional (3D) bioprinting. Micromachines (Basel).

[109] Gungor-Ozkerim PS, Inci I, Zhang YS, Khademhosseini A, Dokmeci MR (2018). Bioinks for 3D bioprinting: an overview. Biomater Sci.

[110] Taghizadeh M, Taghizadeh A, Yazdi MA (2022). Chitosan-based inks for 3D printing and bioprinting. Green Chem.

[111] Ng WL, Yeong WY, Naing MW (2016). Development of polyelectrolyte chitosan-gelatin hydrogels for skin bioprinting. Procedia CIRP.

[112] Ng WLK, Yeong WY, Naing MW (2016). Polyelectrolyte gelatin-chitosan hydrogel optimized for 3D bioprinting in skin tissue engineering. Int J Bioprinting.

